# Microfluidics Temperature Compensating and Monitoring Based on Liquid Metal Heat Transfer

**DOI:** 10.3390/mi13050792

**Published:** 2022-05-19

**Authors:** Jiyu Meng, Chengzhuang Yu, Shanshan Li, Chunyang Wei, Shijie Dai, Hui Li, Junwei Li

**Affiliations:** 1 Hebei Key Laboratory of Smart Sensing and Human-Robot Interactions, School of Mechanical Engineering, Hebei University of Technology, Tianjin 300130, China; 201521202004@stu.hebut.edu.cn (J.M.); 201811201004@stu.hebut.edu.cn (C.Y.); 201811201008@stu.hebut.edu.cn (C.W.); 202031205015@stu.hebut.edu.cn (H.L.); 2 State Key Laboratory of Reliability and Intelligence of Electrical Equipment, Hebei University of Technology, Tianjin 300401, China; 3 School of Health Sciences and Biomedical Engineering, Hebei University of Technology, Tianjin 300401, China

**Keywords:** heat transfer, microfluidics, liquid metal, measurement, temperature monitoring, PCR

## Abstract

Microfluidic devices offer excellent heat transfer, enabling the biochemical reactions to be more efficient. However, the precision of temperature sensing and control of microfluids is limited by the size effect. Here in this work, the relationship between the microfluids and the glass substrate of a typical microfluidic device is investigated. With an intelligent structure design and liquid metal, we demonstrated that a millimeter-scale industrial temperature sensor could be utilized for temperature sensing of micro-scale fluids. We proposed a heat transfer model based on this design, where the local correlations between the macro-scale temperature sensor and the micro-scale fluids were investigated. As a demonstration, a set of temperature-sensitive nucleic acid amplification tests were taken to show the precision of temperature control for micro-scale reagents. Comparations of theoretical and experimental data further verify the effectiveness of our heat transfer model. With the presented compensation approach, the slight fluorescent intensity changes caused by isothermal amplification polymerase chain reaction (PCR) temperature could be distinguished. For instance, the probability distribution plots of fluorescent intensity are significant from each other, even if the amplification temperature has a difference of 1 °C. Thus, this method may serve as a universal approach for micro–macro interface sensing and is helpful beyond microfluidic applications.

## 1. Introduction

In recent decades, advances in microfabrication techniques have led to the development of a wide variety of microfluidic devices [1,2,3]. For many microfluidic applications, the knowledge of the temperature field is of high technical and scientific importance [4,5]. However, it remains challenging to obtain accurate temperature data of the microfluid limited by size effects, such as manufacturing process and sensor accuracy [6,7]. Conventional high-precision types of equipment or temperature sensors suffer from expensive costs, professional operation, and poor interference immunity. With the development of micro-electro-mechanical systems (MEMS), while numbers of research on the measurement and calibration of microfluidics temperature have been conducted [8,9,10], a unified and systematic theory is still ambiguous. Therefore, the study on the microfluidic heat transfer characteristics and temperature calibration is kept hot [11,12,13].

Two main methods have been reported for the microfluidics temperature measurement [14,15,16]: direct contact and non-direct-contact temperature measurement. Generally, the temperature can be accurately recorded, but the inherent temperature of the targeted micro fluids will be affected via external macro instruments by contact measurement. There is no heat exchange via measurement tools of the non-contact method, which make it difficult to achieve high accuracy by utilizing the changes in the physical properties of the target object.

For contact temperature measurement, the heterojunction structure of thermocouples with micro or even nano-size can be directly applied to the thermometry of microfluid [17,18,19]. However, we must suffer from damage and vibration when operating such a thin and cuspate thermocouple probe, which does not meet the requirements of high reliability and ease of use. In addition, micron or sub-micron platinum film thermal resistance is also used as an essential method for microfluidic temperature measurement [20,21]. However, it must be attached to a substrate, so it is not suitable for measuring the temperature of a small volume. As active devices, their self-heating effect caused by power may influence the micro-scale temperature distribution. The emerging carbon nanotube technology measures the volume expansion of gallium in carbon nanotubes to read temperature changing [22,23], similar to a micro-nano-scale mercury thermometer. It can be applied to microfluidic temperature measurement, but the accuracy is not high.

Due to the limit of resolution, the mainstream non-contact infrared thermal imaging temperature measurement technology cannot be directly used for microfluidic temperature measurement [24]. In recent years, optical imaging technology has been a powerful means to explore the temperature distribution of microfluidics [25,26]. For example, the temperature change can be characterized by the fluorescence intensity of temperature-sensitive fluorescent dye added to the microfluid [27]. However, reference image comparison is required, and there is considerable system uncertainty, which is susceptible to the effects of cross-color in the optical path. In addition, quantum dots have become a kind of optical temperature measurement materials with development potential due to their advantages of small size, good light stability, and high plasticity [28]. However, its size and shape distribution depending on temperature will cause uneven light emission. In addition, the polymer polyacrylamide can be used as a measure of micro-scale temperature due to its structure variation with temperature [29]. The temperature sensing range can be adjusted by combining reactive polymers with fluorophores. However, polymer-based measurement is relatively slow and unsuitable for real-time temperature monitoring.

In recent years, more and more microfluidic heat transfer principles have been explored and studied to use indirect methods to invert the microfluidic temperature [30]. For example, winding copper wire around a microfluidic pipe as a temperature change resistor to measure microfluidic temperature [31], but the effect of heat transfer from the pipe material is not considered. Color-changing temperature-sensitive materials can monitor microfluidic temperature changes [32], but it is generally effective only for a specific temperature value and have a very narrow range of application. Additionally, liquid metals with low melting points have been widely studied as high thermal conductivity and easy-to-handle materials in microfluidic heat transfer [33]. Liquid metals can be used as thermal conductive media connecting the microscopic and macroscopic to achieve a coefficient of temperature conversion.

In this work, a novel indirect temperature measurement method integrating platinum resistance millimeter-scale industrial sensor and liquid metal has been demonstrated to measure and monitor microfluid temperature in a microfluidic chip channel. With this method, the close contact between the sensor and microfluid is avoided, and it is user-friendly and repeatable. First, the relationship between the chip substrate and the microfluid temperature has been studied. Numerical simulation proves that the temperature of the top surface of the glass substrate is the same as the fluid in the microchannel that contacts it. Therefore, we measure the glass surface temperature to characterize it as the targeted fluid temperature. We then punch holes at the designed position that avoid the microchannels, place temperature sensors to contact the top surface of the glass substrate, then fill liquid metal packaging. The aperture is optimized to achieve the best temperature measurement effect, and the function compensation relationship between the measurement temperature and the microfluid in the range of 30–100 °C is calibrated. In addition, a highly temperature-dependent isothermal amplification PCR experiment was presented to prove the temperature measurement validity of this method. The results show that this measurement and compensation method has great potential in microfluidic temperature monitoring.

## 2. Materials and Methods

### 2.1. Chip Design and Sensor Installation

**Figure 1:**
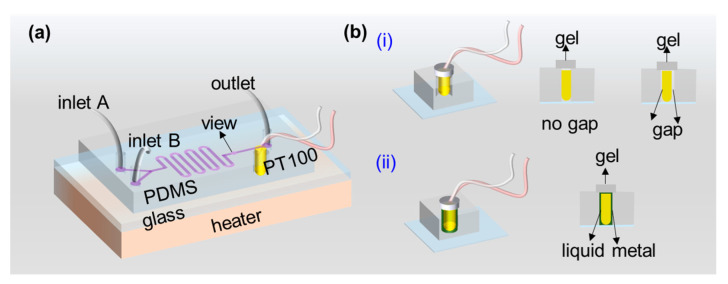
Illustration of the temperature depended on the PCR device, including a piece of PDMS replica bonded with a glass slider, a heater, and a PT100 sensor. (**a**) The full view of our device. The PDMS replica has 2 inlets, 1 outlet, and a through-hole. The PT100 was inserted into the through-hole to measure the temperate of the top surface of the glass slide. The heater was put under the glass substrate. (**b**) Illustration of the packing for PT100 (**i**) w/o and (**ii**) with liquid metal deposited in the through-hole.

Before conducting the numerical optimization studies, the temperature depended PCR chip is performed to validate the physical structure. Here, we present an S-shaped microchannel design that incorporates two inlets introducing two streams for reagent mixing. A through-hole is arranged on the corner, as shown in Figure 1a, which provides the design details of the proposed microfluidic chip. The microchannel consists of a rectangle cross-section channel, 100 μm wide and 30 μm deep, together with an S-shape unit at the central part, which makes the reagent mix more evenly. A circular view area is on the straight microchannel before the exit and one single outlet downstream for postprocessing.

Figure 1b shows the basic concept for sensor installation. First, a sensor is arranged to the hole to ensure its sensitive components contact with the top surface of the glass slide, as shown in Figure 1b(i). Moreover, to ensure that the sensor is in complete contact with the glass surface, the liquid metal is used to fill the gap around the sensor in the hole, as shown in Figure 1b(ii). A relative precise temperature value can be recorded by this method.

### 2.2. Chip Fabrication

Here, the microfluidic channel with suitable patterns is fabricated using standard soft lithography methods previously reported [34,35]. Briefly, the AutoCAD 2018 is used to design the photomasks of the electrode and channel, and then the layout is printed by a commercial high-resolution inkjet printer. Next, the SU-8 photoresist or photosensitive dry film is coated onto the silicon substrate, followed by a pre-bake, exposure, development, as well as the post-bake process to fabricate the mold master. Then, the polydimethylsiloxane (PDMS sylgard 184, Dow Corning) is cast on the mold master and cured at ~80 °C for 1~2 h. After that, the PDMS channel containing the imprint is detached from the mold master and punched to fabricate the inlets, outlets, and through-hole.

The glass slide is soaked in acetone for 20 min, rinsed with isopropyl alcohol and DI water each for 15 s, and then dried with an air gun. PDMS block mentioned above is bonded with a glass surface after plasma treatment by a plasma machine (PDC-MG, PTL Technology Co, Ltd., Shenzhen, China). The plasma treatment and bonding procedure are in four steps: a. Put PDMS block with the channel side exposed and cleaned glass slide into the plasma bonding machine. b. Vacuum the machine to a pressure value of about 100 Kpa. c. Turn on plasma high voltage discharge and last 40 s. d. Take out the PDMS block and bond it to the glass slide in time to avoid surface contamination or denaturation.

### 2.3. Reagent Preparation

The kit for duck-derived gene detection (including dry powder reagent tube, R buffer, B buffer, and positive gene template) was purchased from Anpu Biotech Co., Ltd. (Changzhou, China). The best amplification temperature of this reagent is 39–41 °C, and the reaction time takes 20 min. Before the experiment, it is necessary to add 45 μL R buffer, 2.5 μL positive gene template, and 2.5 μL B buffer to the dry powder reagent tube in sequence. Then, mix the reagents evenly on the rotating table. It should be noted that the entire reagent preparation process needs to be carried out in a fume hood and on an icebox to prevent air pollutants and high temperatures from affecting the reaction system. After the reagents are prepared, store them at 0–4 °C for later use, and keep the remaining reagents in a refrigerator at −20 °C.

### 2.4. Temperature Calibration and Solution Delivery

**Figure 2:**
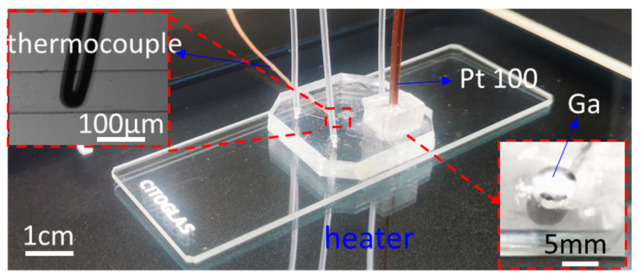
Photo of our microfluidic device mounted on a miniaturized heater. The high-precision smart thermocouple was used to measure the temperature of the fluid within the microchannel. The industrial PT100 sensor inserted into the through-hole was to detect the top surface of the glass slide. Gallium liquid metal was used to fill the gaps between PT100 and the through-hole.

A transparent tempered glass heater (SAPPHIRE SC-6100, TY154, Merip Technology Co, Ltd., Nanjing, China) is used to provide a heat source for the microfluidic chip, so that the bottom surface of the microfluidic chip has a constant temperature, as shown in Figure 2, and the temperature error of the heater is 0.1 °C. The platinum resistance temperature sensor (PT 100-2mm, Sensite, Beijing, China) is encapsulated in the through-hole of the PDMS block with glue together, packaging liquid metal to measure the temperature of the upper surface of the glass slide and record the data, as shown in Figure 2. In addition, a high-precision thermocouple temperature sensor (tt-k-40-36, Omega, New York, NY, USA) is packaged inside the microchannel to measure and record the temperature data of the microfluid. Then, we make a compensation function for the temperature relationship between the two mentioned above. According to the function calculation, the glass surface temperature can be used to characterize the temperature of the microfluid, avoiding the use of precision thermocouples.

In order to quantify the performance of the temperature measurement, a PCR amplification experiment that was highly dependent on the temperature is present. Here, two streams of aqueous reagent are pumped through the inlets with separate, independently controlled programmable flow pumps (PC1, Elveflow, Paris, France) to form a laminar flow at the very beginning of the chip. Then laminar fluids flow through the S-shaped channel, ensuring more effective reagent mixing [36]. When the fluid fills the channel, the heater is carefully adjusted to a specific temperature and lasts 20 min. Then observe the fluorescence effect by a microscope (Eclipse Ti-s, Nikon, Tokyo, Japan) in the view area downstream of the microchannel.

## 3. Numerical Analyses

### 3.1. Control Equation and Boundary Condition Settings

The physical field modules of the heat transfer in solids and fluids and events are used for simulation.

#### 3.1.1. Heat Transfer in Solids and Fluids

Energy conservation equation:

It is assumed that it is all heat transfer between solid and fluid, the energy conservation equation is given by,

(1) $$\rho VC_{p}\frac{\partial T}{\partial t}+\nabla \cdot \left(-Vk\nabla T\right)=Q_{0}$$

where ρ is the density of the applied material, V is the volume of the heated object, $C_{p}$ is the capacitance measured at constant pressure, k is the thermal conductivity, $Q_{0}$ is the energy generation rate for the whole system.

In order to simulate the temperature control of the heater, a constant power $p_{0}$ and a status indicator StateHeater (set in Event interface) are selected as thermal energy source, the energy generation rate is set as,

(2) $$Q_{0}=p_{0}\times \mathrm{StateHeater}$$

It is assumed that the bottom of the heater is thermal insulation, the boundary condition can be set as,

(3) n·(−k∇T)=0

It is assumed that the outside of the system is heat dissipated by natural convection, the convective heat flux should be given by,

(4) $$n\cdot \left(-k\nabla T\right)=h_{c}\left(T-T_{0}\right)$$

where $h_{c}$ is the natural convection heat flux coefficient, $T_{0}$ is the ambient temperature.

The radiant heat flux of the system to the ambient can be set as,

(5) $$n\cdot \left(-k\nabla T\right)=\varepsilon \sigma \left(T^{4}-T_{0}^{4}\right)$$

where ε is the emissivity of the material, 0 < *ε* < 1, σ is the Stefan–Boltzmann constant.

There is contact heat loss between the heating block and the glass slide, which mainly includes contact and gap thermal resistance, which can be expressed as

(6) $$n\cdot \left(-k\nabla T\right)=h\left(T_{down}-T_{up}\right)+rQ_{0}/A_{l}$$

where *r* is the heat partition coefficient.

(7) $$h=h_{contact}+h_{gap}$$

The heat transfer coefficient of thermal contact resistance is closely related to the surface roughness, microhardness, and contact pressure of the material. It should be expressed as [37],

(8) $$h_{contact}=1.25k_{contact}\frac{m_{asp}}{\sigma_{asp}}{\left(\frac{P}{H_{c}}\right)}^{0.95}$$

where $k_{contact}$ is the thermal conductivity of materials in contact, $m_{asp}$ and $\sigma_{asp}$ are the asperities average slope and height surface roughness, respectively. *P* is contact pressure between the heater and glass slide. $H_{c}$ is the microhardness of the glass.

The gap thermal resistance is related to the gas type and contact pressure between two contacting objects. Still, there is no specific expression function yet, and the value range can be checked.

#### 3.1.2. Events

The Event interface is used to control the temperature of the heater to simulate closed-loop PID control. First, the steady-state error is defined as *T*_*error*_ = 0.1 K. First, the discrete state of the Event is defined as StateHeater = 1, and the given power $Q_{0}$ = $p_{0}$ × StateHeater, then heating starts. When heating to the upper limit of the target temperature, *T* > *T*_*error*_ + *T*_*target*_, at this time StateHeater = 0, stop heating. When the temperature drops to *T*_*t*_ < *T*_*targe*_ − *T*_*error*_, the heater starts working again, StateHeater = 1. The state function can be set as,

(9) $$\mathrm{StateHeater}=\{\begin{array}{c} 1\;T\leq T_{target}+T_{error}\;\left(1\right)\\ ↓\\ 0\;T>T_{error}+T_{target}\;\left(2\right)\\ ↓\;↑\\ 1\;T<T_{target}-T_{error}\;\left(3\right) \end{array}$$

**Figure 3:**
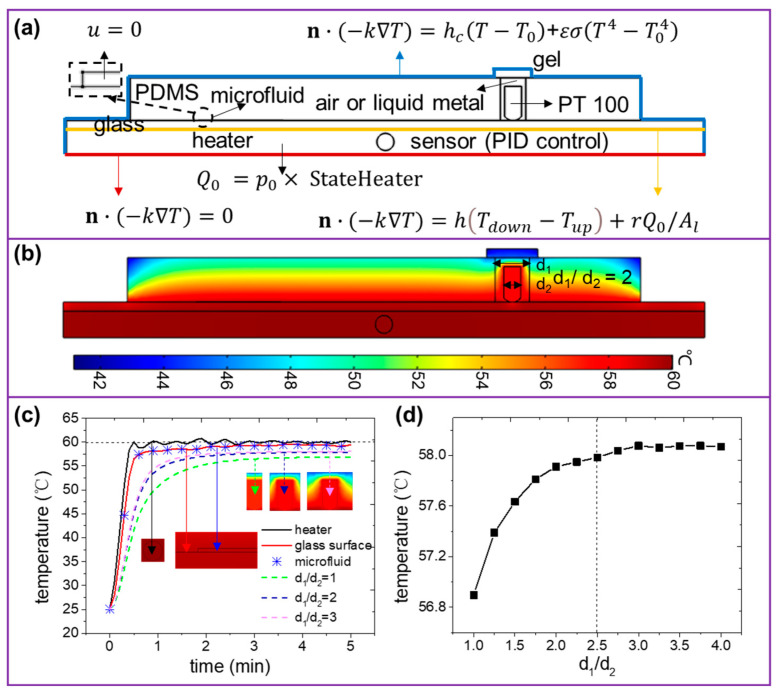
(**a**) Boundary condition settings of the microfluidic heating system. (**b**) The temperature field of the microfluidic heating system. Here the target temperature is 60 °C and d_1_/d_2_ = 2. (**c**) The real-time temperature of the heater, glass, and microfluid with different hole sizes. Here the heating power is 30 W. (**d**) The experimental steady-state temperature as a function of d_1_/d_2_.

After state (1) reaches its peak, states (2) and (3) act cyclically to stabilize the temperature. Feedback temperature is taken from the integral temperature of the circular sensor on the heater, as shown in Figure 3a. The simulation domain and boundary condition settings are shown in Figure 3a.

### 3.2. Parameter Settings in the Simulation

Since the structure of the 3D model is relatively regular and has similar vertical sections, here we did not propose an actual 3D model to calculate the heat transfer process. For simplicity, we presented a longitudinal cross profile 2D numerical model herein. The heat transfer model is conducted using commercial finite element software COMSOL Multiphysics 5.4. The model discretization and grid independence, as well as convergence solving methods, are shown in the Supplementary Material, including a discrete grid for the simulation model (Figure S1), numbers of the domain and boundary elements in different element sizes (Table S1) and grid independence verification (Figure S2). The physical and geometry parameters in our study are set as the same as those in experiments, as listed in Table S2 in Supplementary Materials.

## 4. Results and Discussion

### 4.1. Effects of Aperture on Temperature Measurement

The temperature signal measurement by platinum (Pt) resistance sensor is contact conduction via energy. The heat transfer capacity for ambient materials placed adjacent to the sensor has a significant influence on receiving feedback signals. First, we put the Pt sensor directly into the hole, get as close as possible to contact the top surface of the glass slide, then seal it with gel. For instance, making the aperture of the hole equal to twice that of the sensor, and setting the target temperature is 60 °C. The numerical result shows the temperature distribution of the 2D microfluidic chip heat transfer system in Figure 3b. It can be seen that the temperature gradient on the heater is not visible to the naked eyes due to the excellent performance for heat transfer of aluminum products. The temperature distribution on the vertical profile of the glass slide presents a slight bottom-up temperature gradient and a lower overall temperature than the heater. Two reasons cause the loss of energy: convection heat dissipation between the top surface of the glass slide and the ambient soft-temperature air; contact heat resistance between the bottom surface and the heater. Further, the microfluidic temperature, on which visual appearance, is almost the same as that of the top surface of the glass slide. However, the temperature traversing the vertical profile of the PDMS block decrease clearly and gradually from bottom to top due to the character of low thermal conductivity of PDMS and great convective heat flux presented on the top layer. The temperature appears to be the weakest at the corners caused by concurrent heat flux on both the top and side surfaces. In addition, the temperature measured by the Pt sensor on the top surface of the glass slide is almost uniform and slightly lower than that covered by the PDMS block. The intrinsic heat conduction of the Pt sensor, air, and sealing gel results in a decrement in temperature.

The heat transfer time-dependant process can be decomposed as follows. First, the temperature of the heater rises to the steady-state status within 1 min heating by 30 W power with ±0.1 °C error, as shown by the black solid curve in Figure 3c, which is consistent with the control method utilized in the experiment. The temperature of the top surface of the glass slide rises to the stable target later than that of the heater due to the thermodynamic hysteresis effect, as shown by the solid red curve in Figure 3c. It almost simultaneously reaches a stable status compared to microfluidic temperature with the top surface temperature of the glass, and the difference in the steady value is negligible, as shown by the blue star marks in Figure 3c. Therefore, the microfluidic temperature can be an equivalented substitute to that of the top surface of the glass slide. The Pt resistance sensor is used to sense the temperature of the top surface of the glass slide by packaging it in a cylindrical hole through the PDMS block. Actually, the feedback temperature via the Pt resistance sensor is later and lower than that of the top surface of the glass slide. The stable temperature changes regularly with the aperture, as shown by dotted curves in Figure 3c. When the aperture (d_1_) is equal to the diameter of the Pt resistance sensor (d_2_), that is d_1_/d_2_ = 1, the pieces of the PDMS block in contact with the Pt resistance sensor carry away the heat making the temperature measured by the sensor much less than that of the top surface of the glass slide. The feedback temperature of the Pt resistance sensor gradually increases as the aperture duo to the contact between the sensor and PDMS has been blocked by air with relatively high thermal conductivity blocks. The feedback temperature is not monotonically increasing with the continued expanding aperture but will eventually stabilize at a certain value, as shown in Figure 3d. This phenomenon is caused by the balance of heat transfer and dissipation around the sensor. Since the error value is ±0.1 °C, the measured temperature of the sensor will reach a stable value when d_1_/d_2_ = 2.5. The average steady-state temperature is calculated as 58.08 °C. In order to facilitate the hole processing and sensor packaging in the experiment, take d_1_/d_2_ = 3 for experimental research.

### 4.2. Liquid Metal Filled and Experimental Temperature Calibration

**Figure 4:**
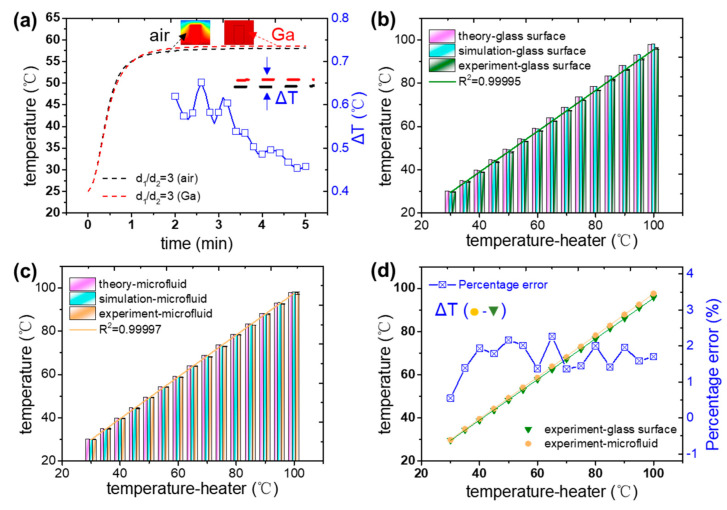
(**a**) The temperature (left *y*-axis) and temperature difference (right *y*-axis) as a function of time, with the condition of with and without liquid metal filled in the gap. Here d_1_/d_2_ = 3. The theoretical, simulated, and experimental temperature of glass surface (**b**) and microfluid (**c**) with the set temperature in a range of 30–100 °C. (**d**) The comparison of experimental temperature between the glass surface and microfluid. Here the average temperature error is 1.74%.

It is hard to make a Pt resistance sensor repeatedly be placed closely in a giant hole, which should affect the accuracy of the feedback signal. In order to ensure that the sensitive elements of the sensor are entirely in contact with the top surface of the glass slide, meanwhile, away from the PDMS block, liquid metal with high thermal conductivity and flexibility is used to fill the gap between the cylindrical hole and the Pt resistance sensor. In this way, the measurement accuracy of the sensor gets significant improvement. As shown in the double *y*-axis plot in Figure 4a, when the hole is, the temperature distribution of the sensor has become uniform with liquid metal filled in the gap, and the steady-state temperature is higher than that of air that exists. When the steady-state temperature is reached, the average measured temperature difference between the filled liquid metal and air medium is about 0.5 °C, which is five times the allowable error.

There were three times experimental measures were performed to record the steady-state temperature value of the top surface of the glass slide in five different locations within the range of 30–100 °C. The average temperature and variance are shown in Table 1.

The difference value between the top surface of the glass slide measured by Pt sensors and the set temperature gradually increases from 30 °C to 100 °C, as shown by the green column in Figure 4b. The measured temperature has a linear relationship with the set temperature of the heater (R^2^ = 0.99995), within the range of 30–100 °C. The linear fitting relationship can be expressed as

(10) $$T_{act}=0.94T_{set}+1.35$$

where $T_{act}$ is the measurement temperature of the top surface of the glass slide, $T_{set}$ is the set temperature.

At the unperforated position, the temperature of the top surface of the glass slide changes with the set temperature in simulation, as shown by the cyan column in Figure 4b. The simulated results are almost the same as the theoretical values but greater than the measured by the experiment, as shown in Figure 4b. It means that under the same set temperature, the temperature of the top surface of a glass slide that is unperforated is higher than that of the sensor location. It is consistent with the law of steady-state temperature difference calculated by the simulation mentioned above when the target temperature is 60 °C. In order to further determine the relationship between the temperature of the glass surface at the perforation and that of the microfluid, the temperature of the microfluid was measured by placing a precision thermocouple in the microchannel. The average temperature value and variance of the three measurements are shown in Table 2.

The results are highly consistent with the simulated top glass surface temperature where unperforated, as shown in Figure 4c, which further illustrates the accuracy of the measurement and the rationality of the glass surface temperature characterizing the microfluidic temperature. In addition, according to the theoretical steady-state heat transfer of the multilayer medium, the heat transfer rate can be expressed as [38]

(11) $$q=\frac{T_{set}-T_{0}}{\left[1/\left(h_{1}A_{1}\right)+L_{i}/\left(k_{i}A_{i}\right)+R_{cont}+1/\left(h_{i+1}A_{i+1}\right)\right]}$$

where $h_{1}$ and $h_{i+1}$ are the convective heat-transfer coefficient of the bottom and top, respectively. *A* is the surface area of the object with heat flux. $L_{i}$ and $k_{i}$ are the thickness and thermal conductivity of each layer (*I* = 2, 3,…), respectively. $R_{cont}$ is the contact resistance between heater and glass slide, $R_{cont}$ = 0.3–0.6 × 10^−4^ m^2^∙K/W.

Assuming that each layer constructed in the microfluidic system could transfer heat uniformly in the vertical profile, and take the bottom of the heater as the zero point, the temperature of the upper layers can be expressed as:

(12) $$T\left(y\right)=T_{set}-q\frac{y}{k_{i}A_{i}}$$

where *y* is the distance from the bottom surface of the heater.

According to Equation (12), the relationship between the temperature of the top surface of the glass slide and the set temperature can be calculated as shown by the pink column in Figure 4b. The theoretical analyses are extremely consistent with the simulation and experimental results. Therefore, we can analyze the relative temperature relationship between the top surface of the glass slide measured by the Pt 100 resistance sensor at the hole punching and the microfluid. As shown by the blue curve in Figure 4d, the difference between the microfluidic and measured temperature at each temperature is mostly concentrated between 1% and 2%. Excluding the more significant error at 30 °C, the average error is 1.74%. Therefore, the temperature function between the microfluid and the heater can be expressed as

(13) $$T_{micro}=T_{act}\times \left(1+1.74\%\right)=0.96T_{set}+1.37$$

The microfluidic temperature value can be derived from the temperature setpoint without precision sensor measurements in this system. Even though it would be desirable to place a temperature sensor as close as possible to the location of the microfluidic, practical limits usually prevent this, such as limited space and tight bonding requirements. The emergence of precision instruments can break through the limitations of assembly space but still suffer from damage and expensive cost. On the other hand, customized MEMS sensors integrated on a microfluidic chip may lead to unexpected contaminations into liquid samples. Here, a millimeter-scale industrial grade temperature sensor is introduced for microfluidic temperature sensing, and the compensation relationship between the microfluids and the glass substrate is investigated. As a more reliable and low-cost sensor, Pt100 is also capable of providing industrial-grade precisions. It also establishes a link between micro and macro scale, which enriches the selectivity of external control devices. Although calculations are required, the optimized relational equation can be embedded inside the temperature control system to achieve a one-step operation in the future. When the substrate material is changed, the microfluidic temperature value can still be calculated from the upper surface temperature value of the substrate.

### 4.3. Temperature-Dependent PCR Experiment

**Figure 5:**
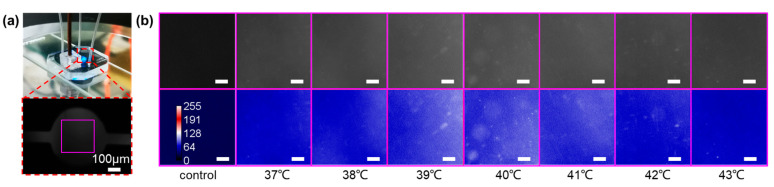
(**a**) The microfluidic chip for PCR tests with the microscopic image of a microchamber. (**b**) Top: fluorescent images of control and positive reagents at 37–43 °C. Bottom: blue pseudo-color images of the top. The scale bar is 50 μm.

A temperature-dependent PCR experiment is conducted to test the accuracy of the temperature measurement method described above. The reaction reagents are pumped into the microfluidic chip and then placed the chip on the heater to stay for 20 min. At a suitable temperature, the DNA template, primers, and enzymes in the reaction reagents interact to complete the unwinding and replication of the double-stranded strands. Concurrently, the fluorophores are activated so that the reacted sample exhibits a fluorescent effect. According to the fluorescence intensity, the content of the target DNA in the original sample can be calculated. The sample kit indicates that the best temperature for reagent amplification is between 39–41 °C. In order to explore the effect of temperature on the amplification effect, we set low and high temperatures relative to the optimal value for isothermal amplification of sample reagents. After the reaction is completed, the fluorescence of the reagent is observed through a microscope. The blue light is selected as the excitation light, and the fluorescence diagram of the view area is shown in Figure 5a. A 300 μm square centrally inside the view area (d = 500 μm) is cut out as the analysis area for fluorescence intensity to avoid the error caused by the edge of the microchannel. As shown in the top part of Figure 5b, the results show the fluorescence graphs of the isothermal amplification PCR with a microfluid temperature of 37–43 °C. The temperature measured by the sensor is 36.4 °C, 37.4 °C, 38.3 °C, 39.3 °C, 40.3 °C, 41.3 °C, and 42.3 °C. In order to obtain a consuming contrast, grey-level images are processed with pseudo-color, as shown in the bottom part of Figure 5b. The results show that the fluorescence intensity at 39–41 °C is higher than that at 37 °C, 38 °C, 42 °C, and 43 °C, which is consistent with the informed. The accuracy and practicability of the method mentioned above for temperature measurement are explained. The reason for the varying fluorescence intensity in the graphs is mainly due to the inevitable precipitation in the reactants, which makes the fluorescence intensity of some areas increase locally. Another reason is speculated as to the thickness error in the chip manufacturing process. Hence, we can analyze the difference in fluorescence intensity of each group through the overall surface average fluorescence data.

**Figure 6:**
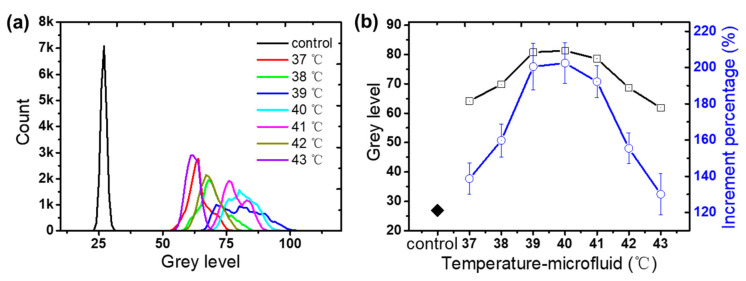
(**a**) Probability distribution of grey scales from 0 to 255. (**b**) Weighted average grey level of each image and increment percentage to control group against the different temperatures of microfluid.

The average surface grey level of each picture is calculated, which is used to represent the fluorescence intensity. Each picture contains 20,736 pixels. The distribution of surface grey levels (0–255) at different temperatures is shown in Figure 6a. It can be seen that the grey level of the control group is the smallest and most concentrated. The rest of the distribution is also obviously discriminative at each temperature, and it shows a trend of first increasing and then decreasing. In order to further quantify the raw data, the weighted average of each grey band is calculated, as shown in the black curve of Figure 6b. The grey level reaches the maximum at 40 °C, and it has a slight distinction at 39–40 °C. Three experiments were performed to verify the reliability of this measurement method. The percentage increase in the average fluorescence intensity of the fluorescence group relative to the control was calculated as shown in the blue curve in Figure 6b.

The percent of fluorescence intensity increasing at different temperatures is shown in Table 3. These results show that this measurement method has a good ability to distinguish temperature variation. In previous studies, temperature sensors were placed outside the microfluidic area similar to our method, such as, next to the microfluidic device [39], together with the heater [40], in the reference position [41]. However, the issue in these cases is the precise inside temperature cannot be guaranteed. Therefore, the compensation relationship between the microfluids and the substrate is investigated in this work. In other research, one approach to reducing temperature errors is modifying the control method [42]. Unfortunately, the complex circuit systems and electrode fabricated increase uncertainty. The nanophotonic sensor [43] embedded in a microfluidic chip reaches high precision but is customized. As a more reliable and low-cost sensor, industrial-grade temperature sensors used in our model are capable of providing industrial-grade precisions suitable for various devices to reuse.

## 5. Conclusions

Here in this work, a heat transfer model was presented to investigate the relationship between the microfluids and the glass substrate of a typical microfluidic device. With an intelligent structure design and liquid metal, the millimeter-scale industrial temperature sensor could be utilized for temperature sensing of micro-scale fluids. The method overcomes the limitations of temperature sensing for microfluids. The dynamic linear range of measured temperature is demonstrated from 30 °C to 100 °C, and the uncertainty error is below 0.5 °C. Further, temperature-sensitive nucleic acid amplification experiments have been conducted to clarify the temperature resolution of this method. Therefore, it can be surmised that this method shows high potential for micro–macro interface sensing and is helpful beyond microfluidic applications.

## Figures and Tables

**Table 1:** The steady-state temperature value of the top surface of the glass slide.

Set Temperature (°C)	Glass Surface Temperature (°C)
30	29.67 ± 0.06
35	34.40 ± 0.26
40	38.80 ± 0.17
45	43.50 ± 0.10
50	48.17 ± 0.25
55	53.03 ± 0.15
60	57.83 ± 0.12
65	62.40 ± 0.17
70	67.27 ± 0.15
75	71.97 ± 0.12
80	76.67 ± 0.21
85	81.53 ± 0.35
90	86.17 ± 0.38
95	91.03 ± 0.35
100	95.9 ± 0.4

**Table 2:** The steady-state temperature value of the microfluid.

Set Temperature (°C)	Glass Surface Temperature (°C)
30	29.83 ± 0.02
35	34.88 ± 0.06
40	39.57 ± 0.08
45	44.29 ± 0.06
50	49.24 ± 0.12
55	54.13 ± 0.07
60	58.64 ± 0.12
65	63.85 ± 0.06
70	68.19 ± 0.06
75	73.02 ± 0.18
80	78.23 ± 0.07
85	82.71 ± 0.02
90	87.88 ± 0.19
95	92.5 ± 0.17
100	97.56 ± 0.59

**Table 3:** The percent of fluorescence intensity increased.

Set Temperature (°C)	Fluorescence Intensity Increased (%)
37	138.69 ± 8.51
38	159.82 ± 9.09
38	200.48 ± 12.84
40	202.46 ± 11.11
41	192.22 ± 8.80
42	155.43 ± 8.51
43	130.10 ± 11.40

## Data Availability

The data that support the findings of this study are available from the corresponding author, upon reasonable request.

## Supplementary Materials

The following supporting information can be downloaded at: https://www.mdpi.com/article/10.3390/mi13050792/s1, Table S1: Numbers of the domain and boundary elements in different element sizes. Table S2: Parameter settings in the fluid-structure interacted simulation model. Figure S1: Discrete grid for the simulation model. Figure S2: Grid independence verification.
