# Supplementary material for: Microfluidics Temperature Compensating and Monitoring Based on Liquid Metal Heat Transfer
Source: Micromachines (Basel). 2022 May 19;13(5):792. doi: 10.3390/mi13050792 (PMC9146403; doi:10.3390/mi13050792)
Supplement: Supplementary file 1 [file micromachines-13-00792-s001.zip › micromachines-1722178-supplementary.pdf]

Microfluidics Temperature Compensating and Monitoring Based on Liquid Metal Heat Transfer

1. The grid discretization/ independence and convergence solving

Thanks for your point. The finite element (FEM) software COMSOL Multiphysics 5.4 is used for simulation. In this model, free triangle units are employed as domain elements due to the excellent shape adaptation characteristics; the corresponding diagram is shown in Figure S1. The domain elements around the microfluid and sensor are denser than overall, thus achieving accurate calculation of the temperature.

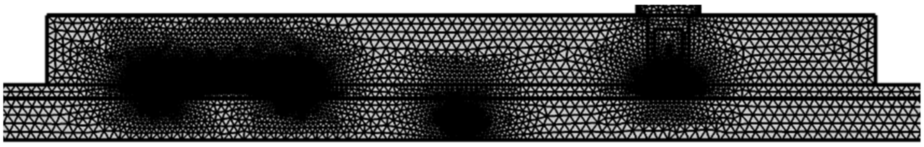

Figure S1. Discrete grid for the simulation model.

According to the physical field, the model is solved using the BDF (backward difference formula) method, by which accuracy varies between first and fifth orders. The default CFL number is set as 5 in this model. For the convergence criterion, the iteration was stopped until the solution reduces to within the desired tolerance. The relative tolerance of this model is set as 0.01.

Regarding the grid division independence, the physical-controlled division method is chosen. The statistical results of different grid "domains" and "boundaries" are shown in Table S1. To optimize the grid selection, we investigated the relationship between the steady-state temperature of the glass surface and the number of domain elements, as shown in Figure S2. When the numbers of domain elements reach 9251, the steady-state temperature of the glass surface almost no longer changes. So we choose 9251 grids for model discretization.

Table S1. Numbers of the domain and boundary elements in different element sizes.

Element sizes	Domain elements	Boundary elements
extremely coarse	3094	580
extra coarse	3491	606
coarser	4566	676
coarse	5272	683
normal	6477	735
fine	7752	784
finer	9251	830
extra fine	14687	917
Extremely fine	19539	1119

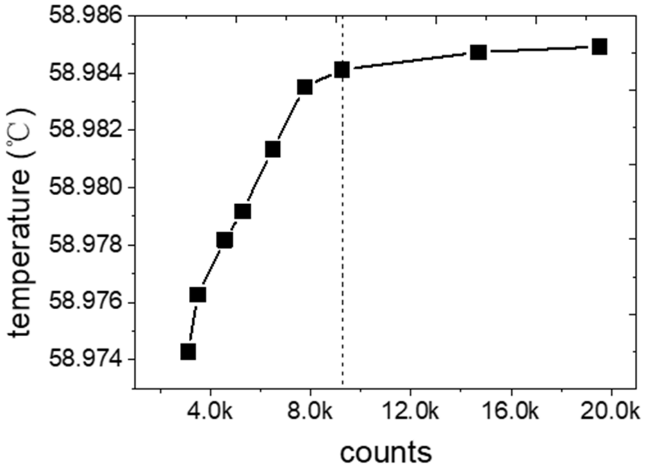

Figure S2. Grid independence verification.

2. The physical and geometry parameters in our study are set as in Table S2.

Table S2. Parameter settings in the fluid-structure interacted simulation model.

parameters	value	unit	description
ρ_{Al}	2.7 x 10^3	[kg/m^3]	The density of the heater and sensor
ρ_{glass}	2.5 x 10^3	[kg/m^3]	The density of the glass slide
ρ_{fluid}	1000	[kg/m^3]	The density of the fluid
ρ_{air}	1.293	[kg/m^3]	The density of the air
ρ_{Ga}	5.9 x 10^3	[kg/m^3]	The density of the liquid metal
k_{Al}	237	[W/(m·K)]	The thermal conductivity of the heater and sensor
k_{glass}	0.77	[W/(m·K)]	The thermal conductivity of the glass slide
k_{fluid}	0.598	[W/(m·K)]	The thermal conductivity of the fluid
k_{air}	0.0267	[W/(m·K)]	The thermal conductivity of the air
k_{Ga}	40.6	[W/(m·K)]	The thermal conductivity of the liquid metal
C_{p_Al}	0.88	[kJ/(kg·K)]	The specific heat capacity of the heater and sensor
C_{p_glass}	0.837	[kJ/(kg·K)]	The specific heat capacity of the glass slide
C_{p_fluid}	4.184	[kJ/(kg·K)]	The specific heat capacity of the fluid
C_{p_air}	1.005	[kJ/(kg·K)]	The specific heat capacity of the air
C_{p_Ga}	0.37	[kJ/(kg·K)]	The specific heat capacity of the liquid metal
η_{fluid}	1.08e^{-3}	[Pa·s]	The viscosity of the fluid
η_{Ga}	1.6e^{-3}	[Pa·s]	The viscosity of the liquid metal

p_0	30	[W]	Heating power
h_c	10	[W/(m^2·K)]	Natural convective heat transfer coefficient
ε	0.8		The emissivity of the material
m_{asp}	0.4		The asperities average slope surface roughness
σ_{asp}	5	[μm]	The asperities average height surface roughness
P	53.3	[N/m^2]	The contact pressure between the heater and glass slide
H_c	3	[GPa]	The microhardness of glass
h_{gap}	10	[W/(m^2·K)]	Gap thermal resistance heat transfer coefficient
r	0.7		The heat partition coefficient
d_1	2	[mm]	The diameter of the PT 100 sensor
d_2	n x d_1	[mm]	The diameter of the through-hole
T_0	25	[degC]	The room temperature
